# Correlation of Insulin-Like Growth Factor 1 With Cognitive Functions in Mild Traumatic Brain Injury Patients

**DOI:** 10.1089/neur.2023.0085

**Published:** 2023-11-07

**Authors:** Ju-Chi Ou, Yin-Hsun Feng, Kai-Yun Chen, Yung-Hsiao Chiang, Tsung-I Hsu, Chung-Che Wu

**Affiliations:** ^1^ Neuroscience Research Center, Taipei Medical University, Taipei, Taiwan.; ^2^ Department of Surgery, School of Medicine, College of Medicine, Taipei Medical University, Taipei, Taiwan.; ^3^ Chi Mei Medical Center, Tainan, Taiwan.; ^4^ PhD Program in Medical Neuroscience, Taipei Medical University, Taipei, Taiwan.; ^5^ International Master Program in Medical Neuroscience, College of Medical Science and Technology, Taipei Medical University, Taipei, Taiwan.; ^6^ Department of Neurosurgery, Taipei Medical University Hospital, Taipei, Taiwan.; ^7^ National Health Research Institutes, Taipei, Taiwan.; ^8^ TMU Research Center of Cancer Translational Medicine, Taipei Medical University, Taipei, Taiwan.; ^9^ PhD Program in Drug Discovery and Development Industry, College of Pharmacy, Taipei Medical University, Taipei, Taiwan.

**Keywords:** cognitive dysfunction, insulin-like growth factor 1, mild traumatic brain injury

## Abstract

Mild traumatic brain injury (mTBI) is a prevalent health concern with variable recovery trajectories, necessitating reliable prognostic markers. Insulin-like growth factor 1 (IGF-1) emerges as a potential candidate because of its role in cellular growth, repair, and neuroprotection. However, limited studies investigate IGF-1 as a prognostic marker in mTBI patients. This study aimed to explore the correlation of IGF-1 with cognitive functions assessed using the Wisconsin Card Sorting Test (WCST) in mTBI patients. We analyzed data from 295 mTBI and 200 healthy control participants, assessing demographic characteristics, injury causes, and IGF-1 levels. Cognitive functions were evaluated using the WCST. Correlation analyses and regression models were used to investigate the associations between IGF-1 levels, demographic factors, and WCST scores. Significant differences were observed between mTBI and control groups in the proportion of females and average education years. Falls and traffic accidents were identified as the primary causes of mTBI. The mTBI group demonstrated worse cognitive outcomes on the WCST, except for the “Learning to Learn” index. Correlation analyses revealed significant relationships between IGF-1 levels, demographic factors, and specific WCST scores. Regression models demonstrated that IGF-1, age, and education years significantly influenced various WCST scores, suggesting their roles as potential prognostic markers for cognitive outcomes in mTBI patients. We provide valuable insights into the potential correlation of IGF-1 with cognitive functions in mTBI patients, particularly in tasks requiring cognitive flexibility and problem solving.

## Introduction

Traumatic brain injury (TBI) is a significant cause of adult injury-related morbidity and mortality. Its incidence was estimated by the Centers for Disease Control and Prevention to be >1.7 million annually in the United States.^1^ Common acute manifestations of TBI include dizziness, headache, cognitive deficits, and emotional problems (such as emotional lability, depression, and anxiety).^1,2^ Cognitive deficits are often the most disabling and distressing for the affected persons, family members, and society. Cognitive deficits can significantly impair activities of daily living, employment, social relationships, recreation, and active participation in the community.^2,3^

TBI can additionally lead to the development of chronic neurodegenerative disorders, including Alzheimer's disease (AD), Parkinson's disease, frontotemporal dementia, and chronic traumatic encephalopathy.^4,5^ TBI is “a disease process, not an event.” The long-term consequences of the injury need to be considered after initial treatment. The clinical significance of mild TBI (mTBI) lies in its variable and often unpredictable recovery trajectories.^3^ Identifying reliable prognostic markers becomes crucial in assessing individual outcomes and optimizing personalized treatment strategies. The potential involvement of insulin-like growth factor 1 (IGF-1) in neuroregeneration and repair processes makes it an intriguing molecule to investigate in the context of mTBI prognosis.

IGF-1 is a polypeptide that is closely related to the growth and aging of neurons.^6^ A previous study suggests that IGF-1 therapy promotes hippocampus neurogenesis and improves spatial memory in rats.^7^ Additionally, disruptions in growth hormone/IGF-1 signaling have been associated with longevity and age-related conditions, suggesting that IGF-1 may benefit aging, particularly in the brain.^8^ IGF-1 emerges as a pivotal factor in the potential relationship between cognitive function and neurological conditions. Talbot and colleagues establish a link between brain insulin resistance in AD cases and reduced responses to insulin signaling and IGF-1 resistance, contributing to cognitive decline independently of classic AD pathology.^9^ This highlights IGF-1's role in brain function and its impact on cognitive outcomes. Wang and colleagues reinforce the significance of IGF-1 by demonstrating its ability to improve cognitive function and anxiety behavior in rats by activating the phosphoinositide 3-kinase (PI3K)/protein kinase B (Akt)/cAMP-responsive element-binding protein (CREB) pathway, which also mitigates inflammation and oxidative stress in the hippocampus.^6^

These findings underscore the importance of IGF-1 as a neurotrophic factor that plays a crucial role in enhancing cognitive abilities and protecting against neurodegenerative processes. Some studies have also reported alterations in IGF-1 levels post-TBI, with potential correlations between IGF-1 concentrations and recovery outcomes.^10–12^ However, these studies often suffer from small sample sizes and methodological limitations, leaving significant gaps in the current understanding of IGF-1 as a prognostic marker for mTBI.

In this study, we aimed to investigate the association between serum IGF-1 levels and the prognosis of patients with mTBI, utilizing the Wisconsin Card Sorting Test (WCST) to evaluate the cognitive status of patients.^13^ The WCST is a widely accepted neuropsychological assessment tool that evaluates executive functions, including cognitive flexibility, problem solving, and abstract reasoning.^13–16^ By incorporating the WCST, we sought to gain insights into the cognitive aspects of recovery and their potential relationship with IGF-1 levels.

## Methods

### Participants

All participants were recruited from three hospitals of Taipei Medical University (Taipei Medical University, Wan-Fan Hospital, and Shuang-Ho Hospital). This study was approved by the Taipei Medical University–Joint Institutional Review Board (N201512017). Two hundred ninety-five participants agreed to join and completed all questionnaires. The criteria were 20–70 years old, TBI within 2 weeks, Glasgow Coma Scale >12, and loss of consciousness in <30 min. Participants with a history of TBI, epilepsy, or pregnancy were excluded. On the other hand, 200 healthy control volunteers with no history of TBIs were recruited.

### Insulin-like growth factor 1 assay

Blood serum samples were collected and immediately stored at −80°C until subjected to biochemical analysis. Serum IGF-1 was measured using an IGF-1 radioimmunoassay kit (DIAsource ImmunoAssays S.A., Ottignies-Louvain-la-Neuve, Belgium) with a 3.4-ng/mL sensitivity. Intra- and interassay precision variations were <9.1% and <9.0%, respectively.

### Wisconsin Card Sorting Test

Participants were asked to identify a sorting rule, to keep the same rule after a “correct response,” and to change the sorting rule after a “wrong response.” WCST consists of three attributes: shape (triangle, circle, star, and cross); number (1–4); and color (red, blue, yellow, and green). The original WCST consisted of 128 cards, and the shorter version, WCST-64, has all the features of the original WCST except two sets of 64 response cards.^17^ The dependent performance measures recorded were: 1) correct rate; 2) total error (the number of responses that do not match the setting sorting principle); 3) perseverative pesponses (the number of persistent responses to an incorrect characteristic); 4) perseverative error (the number of responses that matches the perseverated-to principle and does not match the presently correct principle); 5) non-perseverative error (the number of incorrect responses that do not match the perseverated-to principle); 6) conceptual-level responses (consecutive correct responses in runs of three or more); 7) number of categories completed; 8) trials to complete the first category; 9) failure to maintain (within a principle, the participants changes response strategies); and 10) Learn to Learn (a positive score indicated improved efficiency).

The sum of the perseverative errors and non-perseverative errors scores was equal to the score of total errors. The initial of the WCST raw scores, initial of the WCST standardized scores, larger score indication, and their variable classification are shown in Table 1.

**Table 1.** Characteristics of the WCST Scores

***Score***	***Raw score abbreviations***	***Large score***	***Standardized score abbreviations***	***Variable classification***
Total correct	TC	Good	—	Continuous
Total error	TE	Poor	TE.T	Continuous
Perseverative response	PR	Poor	PR.T	Continuous
Perseverative error	PE	Poor	PE.T	Continuous
Non-perseverative error	NPE	Poor	NEP.T	Continuous
Conceptual-level response	CLR	Good	CLR.T	Continuous
Categorical complete	CC	Good	—	Continuous
Trial to complete first category	TCC	Poor	—	Continuous
Failure to maintain	FM	Poor	—	Continuous
Learn to Learn	LL	Poor	—	Continuous

WCST, Wisconsin Card Sorting Test.

### Statistical analysis

Descriptive statistics, including mean, the standard deviation for continuous variables, and number (*n*) and percent (%) for categorical variables, were provided. The mean difference between the two groups was compared by Student *t*-tests and chi-square tests for the continuous and categorical variables, respectively. Correlations between continuous variables were assessed by Pearson's correlation coefficient. The association between outcome and multiple variables was evaluated by linear and multi-nomial regression for the continuous and nominal outcomes, respectively. All statistical analyses were performed by the statistical software R (R Foundation for Statistical Computing, Vienna, Austria), and the significant value was set to be 0.05.

## Results

### Participant characteristics and group distribution

Two hundred ninety-five mTBI and 200 healthy control participants were analyzed in this study (see Table 2). The percentage of female participants and the average education years between the two groups were significantly different, but the average age between the two groups was not significantly different (mTBI, 44.30 ± 14.12; control, 44.08 ± 14.96). There were 184 (62.37%) female participants in the mTBI group and 136 (68%) females in the control group. The average education year of the control group was more than that of the mTBI group (control, 14.20 ± 2.60; mTBI, 12.89 ± 3.18). In the mTBI group, 91 (30.85%) and 128 (43.39%) participants were caused by falls and traffic accidents, respectively.

**Table 2.** Demographic Data for mTBI and Health Control (mean [SD])

***Variable***	***mTBI (***n*** = 295)***	***Control (***n*** = 200)***	*p* ***value***
Age, year	44.308 (14.119)	44.075 (14.963)	0.862
Sex Gender, *n* (%)			0.04
Female	184 (62.37)	136 (68)	
Male	111 (37.63)	64 (32)	
Education, year	12.885 (3.181)	14.200 (2.603)	<0.01
Injury mechanism, *n* (%)			—
Falls	91 (30.85)		
Traffic accident	128 (43.39)		
Others	76 (25.76)		

mTBI, moderate TBI; SD, standard deviation.

### Comparison of Wisconsin Card Sorting Test scores between mild traumatic brain injury and control groups

**FIG. 1.**
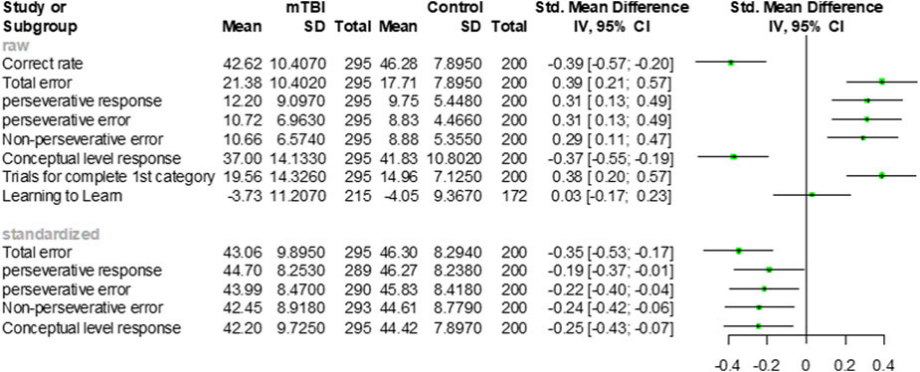
Forest plot of WCST indices. Upper panel: raw score; lower panel: standardized score. CI, confidence interval; mTBI, mild traumatic brain injury; SD, standard deviation; WCST, Wisconsin Card Sorting Test; Std, standardized.

WCST score differences between the mTBI and control groups are shown in Figure 1. Most WCST scores significantly differed between participants in the mTBI and healthy control groups, except the WCST indices, Learning to Learn. The average scores of two WCST indices—correct rate and conceptual level response—in the mTBI group were smaller (worse) than those in the healthy control group. The average raw scores of five indices (total error, perseverative response, perseverative error, non-perseverative error, and trial for completed first category) in the mTBI group were larger (worse) than those in the healthy control group. On the other hand, the average standardized scores of these five indices in the mTBI group were smaller (worse) than those in the healthy control group.

**FIG. 2.**
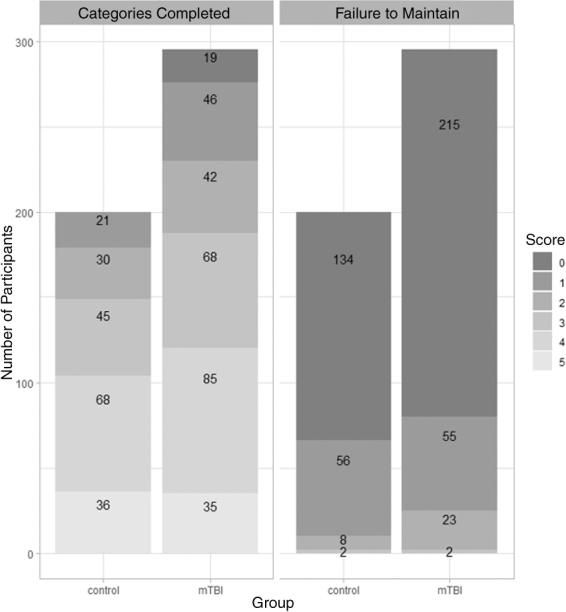
Bar plot for categorical WCST indices (left panel: categories completed; right panel: failure to maintain). WCST, Wisconsin Card Sorting Test.

Bar plots of categories completed and failure to maintain are shown in Figure 2. The number of completed categories ranged from 0 to 5. Eighty-five (28.81%) mTBI and 68 (34%) healthy control participants completed four categories. On the other hand, 19 (8.44%) mTBI participants did not complete any category. For another WCST index (failure to maintain), 215 (72.88%) mTBI and 134 (67%) healthy control participants did maintain successfully without any failure.

### Correlation between Wisconsin Card Sorting Test scores and other variables

**FIG. 3.**
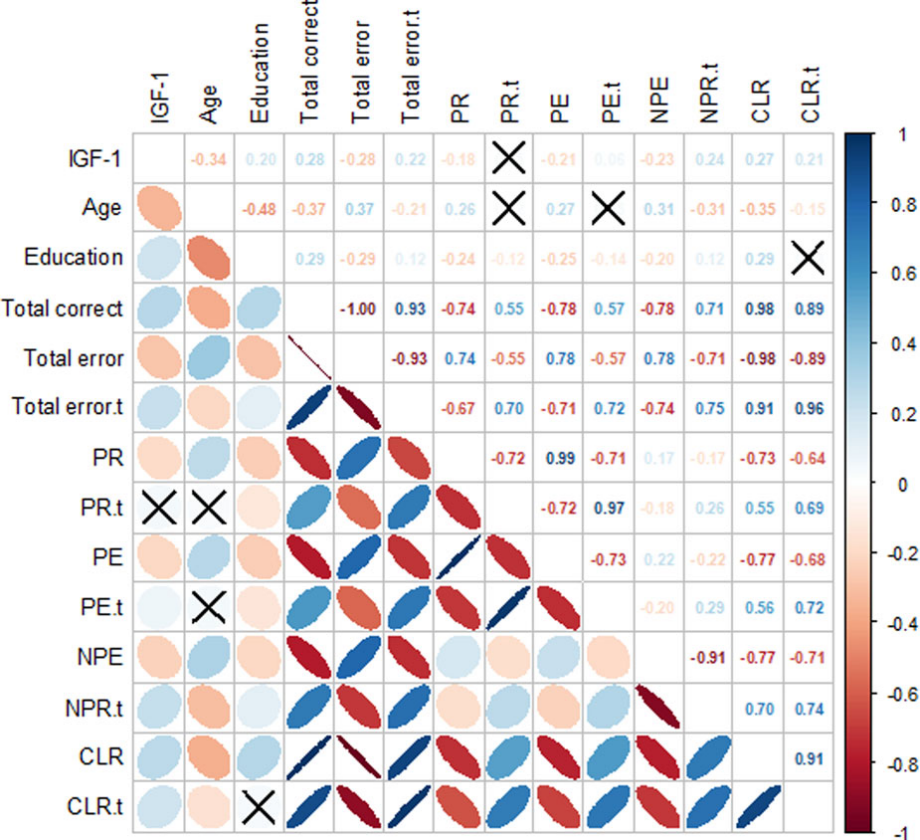
Correlation ellipse matrix (X: not significantly correlated).

Correlation ellipses and tcorrelation coefficients are shown in the upper and lower triangular matrix, respectively (Fig. 3). The colors, shape, and orientation represent the direction and strength of the correlation ranging from −1 (red) to 1 (blue). The standardized scores of perseveration response and perseveration error were not correlated with age and IGF-1, but they were correlated with education year (correlation coefficient = −0.12 and −0.14; *p* < 0.05). The standardized score of the conceptual-level response was not related to education years, but it was correlated with IGF-1 (correlation coefficient = 0.21; *p* < 0.05) and age (correlation coefficient = −0.15; *p* < 0.05). The others were correlated significantly.

### Regression analysis of the Wisconsin Card Sorting Test raw sore

Results of the regression model for each WCST raw score are shown in Table 3. Five risk factors evaluated were group effect (mTBI vs. control), IGF-1, sex, age, and education year. Group and age effects were significant in predicting all five WCST raw scores (correct rate, total error, perseverative response, perseverative error, non-perseverative error, and conceptual-level response). As IGF-1 increased by 1 unit, the correlated rate and conceptual-level response scores increased by 0.019 and 0.025. On the other hand, as age increased by 1 year, these two scores decreased by 0.177 and −0.224. The effects of the education year were significant for all five raw scores except for non-perseverative error. As the education year increased by 1 year, the correct rate and conceptual-level response score increased by 0.312 and 0.473. The score of total error, perseverative response, and perseverative error increased when the IGF-1 level decreased, age increased, or education year decreased.

**Table 3.** Regression Results for WCST Raw Scores

***Linear regression***					
***Estimate (***p ***value)***	***Group (mTBI)***	***IGF-1***	***Sex***	***Age***	***Education***
Correct rate	–2.983 (<0.01)	0.019 (<0.01)	0.800 (0.330)	–0.177 (<0.01)	0.312 (0.039)
Total error	2.983 (<0.01)	–0.019 (<0.01)	–0.805 (0.327)	0.176 (<0.01)	–0.314 (0.037)
Perseverative response	1.941 (0.007)	–0.009 (0.046)	–0.882 (0.216)	0.092 (0.001)	–0.306 (0.019)
Perseverative error	1.469 (0.008)	–0.009 (0.009)	–0.773 (0.159)	0.072 (0.001)	–0.239 (0.018)
Non-perseverative error	1.514 (0.006)	–0.010 (0.005)	–0.032 (0.954)	0.104 (<0.01)	–0.075 (0.457)
Conceptual-level response	–3.845 (0.001)	0.025 (0.001)	0.895 (0.426)	–0.224 (<0.01)	0.473 (0.022)
Trial to complete first category	3.940 (<0.01)	–0.008 (0.229)	1.777 (0.109)	0.133 (0.002)	–0.307 (0.132)
Learn to Learn	0.943 (0.377)	0.011 (0.095)	3.106 (0.004)	–0.018 (0.680)	0.639 (0.002)

***Multi-nomial logistic regression (odds ratio [*p *value])***					
***Odds ratio (*p *value)***	***mTBI***	***IGF-1***	***Male***	***Age***	***Education***
Categorical completed (5-4)	1.199 (0.545)	1.002 (0.239)	0.868 (0.639)	1.020 (0.124)	0.879 (0.051)
Categorical completed (5-3)	1.442 (0.258)	1.001 (0.653)	0.896 (0.734)	1.043 (0.002)	0.857 (0.025)
Categorical completed (5-2)	1.241 (0.551)	0.998 (0.349)	0.724 (0.379)	1.044 (0.004)	0.797 (0.002)
Categorical completed (5-1)	2.173 (0.044)	0.992 (0.007)	0.500 (0.080)	1.067 (<0.01)	0.851 (0.031)
Categorical completed (5-0)	0 participants in control group	0.996 (0.346)	1.591 (0.406)	1.085 (0.001)	0.747 (0.003)
Failure to maintain (3-2)	3.582 (0.252)	0.996 (0.595)	525.206 (<0.01)	0.925 (0.005)	1.228 (0.197)
Failure to maintain (3-1)	1.524 (0.690)	0.997 (0.664)	1624.144 (<0.01)	0.951 (0.063)	1.481 (0.010)
Failure to maintain (3-0)	2.595 (0.363)	0.997 (0.733)	1380.493 (<0.01)	0.942 (0.025)	1.500 (0.007)

WCST, Wisconsin Card Sorting Test; mTBI, moderate TBI; IGF-1, insulin-like growth factor 1.

### Regression analysis of the Wisconsin Card Sorting Test standardized score

Results of the regression model for each WCST standardized score are shown in Table 4. Five risk factors evaluated were group effect (mTBI vs. control), IGF-1, sex, age, and education year.

**Table 4.** Regression Results for WCST Standardized Scores

***Estimate (*** *p* ***value)***	***Group***	***IGF-1***	***Sex***	***Age***	***Education***
Total error	–3.119 (<0.01)	0.019 (0.001)	1.161 (0.173)	–0.105 (0.002)	–0.085 (0.585)
Perseverative response	–2.282 (0.003)	0.007 (0.161)	1.786 (0.021)	–0.023 (0.460)	–0.518 (<0.01)
Perseverative error	–2.534 (0.001)	0.010 (0.042)	2.049 (0.010)	–0.001 (0.983)	–0.541 (<0.01)
Non-perseverative error	–2.221 (0.005)	0.016 (0.002)	0.491 (0.537)	–0.179 (<0.01)	–0.218 (0.136)
Conceptual-level response	–2.373 (0.005)	0.019 (<0.01)	1.206 (0.149)	–0.086 (0.010)	–0.289 (0.060)

WCST, Wisconsin Card Sorting Test; IGF-1, insulin-like growth factor 1.

IGF-1 was a significant risk factor for four WCST standardized scores. As IGF-1 increased by 1 unit, the standardized scores of total error, perseverative error, non-perseverative error, and conceptual-level response increased by 0.019, 0.010, 0.016, and 0.019, respectively. Age was a significant risk factor for three WCST standardized scores. As age increased by 1 year, the standardized score of total error, non-perseverative error, and conceptual-level response decreased by −0.105, −0.179, and −0.086, respectively. Education year is a significant risk factor for two standardized WCST scores. As the education year increased, the standardized WCST score of perseverative response and perseverative error decreased to −0.518 and −0.541.

## Discussion

Herein, we investigated the role of IGF-1 as a potential prognostic marker in mTBI patients. We analyzed data from 295 mTBI and 200 healthy control participants, utilizing the WCST to evaluate cognitive status. Our results revealed significant differences in demographic characteristics between these two groups, with a higher proportion of females and lower average education years in the mTBI group. Notably, falls and traffic accidents were identified as the primary causes of mTBI in our study population. Additionally, the WCST demonstrated pronounced cognitive deficits in the mTBI group compared to controls, highlighting the sensitivity of this neuropsychological assessment tool in detecting mTBI-related cognitive impairments.

In this study, the standardized scores of the perseverative response and perseverative error indices on the WCST were not correlated with age and IGF-1 levels, but they showed significant correlations with education years. This finding suggests that the cognitive flexibility represented by these WCST indices might be less influenced by age or IGF-1 levels, but may be influenced by educational experiences. On the other hand, the standardized score of the conceptual-level response index was correlated positively with IGF-1 levels and negatively with age. This suggests that higher IGF-1 levels might be associated with better conceptual reasoning abilities, essential for problem solving and adapting to changing rules in the WCST. Conversely, advancing age might be associated with reduced conceptual reasoning, a common cognitive decline observed in older persons. Further, regression analyses demonstrated that IGF-1 was a significant risk factor for various WCST raw and standardized scores. As IGF-1 levels increased, scores for the correlated rate and conceptual-level response on the WCST also improved.

These results imply that higher IGF-1 levels might be related to better cognitive functioning, particularly in tasks requiring correct responses and conceptual reasoning. However, it is essential to acknowledge the complexity of the relationship between IGF-1 and cognitive functions.^10,18^ Effects of IGF-1 on cognitive outcomes could be influenced by numerous factors, such as injury severity, time post-injury, and individual variations in IGF-1 responsiveness. Hence, other biological and environmental factors not considered in our study could also affect the IGF-1-cognition relationship.

To gain a deeper understanding of the precise mechanisms underlying the correlation between IGF-1 and cognitive functions, further research is required. Longitudinal studies tracking IGF-1 levels and cognitive outcomes over time, coupled with advanced neuroimaging techniques, could help elucidate the dynamic interactions between IGF-1 and brain function during mTBI recovery. Moreover, experimental studies exploring the specific roles of IGF-1 in neural repair and plasticity processes could offer insights into potential therapeutic targets for improving cognitive outcomes in mTBI patients.^11,18^

The mechanism of IGF-1-rescued cognitive decline after mTBI likely involves the activation of various neuroprotective pathways and modulation of neuronal function in the hippocampus and other brain regions. IGF-1 exerts its effects through the IGF-1 receptor (IGF-1R) and downstream signaling pathways, such as Ras-like without CAAX 1/Akt/SRY-box transcription factor 2 and PI3K/glutamatergic transmission.^19^ Notably, the high expression of IGF-1R in brain regions critically linked to cognition, such as the cerebral plexus, hypothalamus, thalamus, amygdala, and hippocampus/parahippocampal gyrus, underscores the importance of IGF-1 in preserving cognitive function.^20,21^ IGF-1 and IGF-1R deficiencies have been associated with cognitive impairment, further supporting the role of IGF-1 in cognitive recovery after mTBI.^20,21^ Moreover, the upregulation of CREB phosphorylation by IGF-1 and its control of CRE-containing genes, which are vital for neuroprotection and cognitive preservation, suggest a potential mechanism for cognitive recovery.^22,23^ Further, IGF-1 suppresses proapoptotic signals through the mitogen-activated protein kinase/CREB signaling pathway, and the regulation of downstream targets may protect neurons from apoptotic processes and support cognitive function.^24,25^

There are some limitations in this study. First, the recruited participants were mTBI; therefore, an alternative model would be needed to gain the associations and conclusions regarding the effect of more severe TBI. Second, the sole use of the WCST as our cognitive assessment tool might limit the comprehensive evaluation of cognitive functions. Integrating additional cognitive assessments that target different domains, such as memory, attention, and language, would provide a more nuanced understanding of the relationship between IGF-1 and various cognitive outcomes in mTBI. Moreover, measuring IGF-1 levels at a single time point might not fully capture the dynamic fluctuations over the course of mTBI recovery. Multiple measurements over time would offer a more accurate representation of IGF-1 on cognitive outcomes. Further, considering the importance of cognitive assessments, incorporating the Trail Making Test (TMT) could enhance our understanding of cognitive function in mTBI. The TMT assesses executive function and is sensitive to cognitive impairments caused by brain injuries, making it a valuable addition to our cognitive assessment battery.

Recent studies have provided normative data for the TMT across different populations, including patients with TBI, and have highlighted its utility in detecting changes in clinical samples.^26^ Integrating the TMT into future research would contribute to a more comprehensive evaluation of cognitive function in mTBI and its relationship with IGF-1. Addressing these limitations facilitates the development of robust prognostic markers and potential therapeutic interventions for persons affected by mTBI.

Some standardized WCST scores remain associated with age and education level. That means the standardized WCST scores may not be proper for our population, and modification would be needed in future studies. Longitudinal studies are essential to unravel the temporal evolution of IGF-1, cognitive function, and mTBI outcomes over extended periods. Such investigations would allow us to discern the trajectory of cognitive recovery and identify potential windows of opportunity for targeted interventions. Additionally, incorporating multi-modal approaches that include neuropsychological assessments, like the WCST and neuroimaging, fluid biomarkers, and behavioral evaluations, can provide a more holistic understanding of the complex neurobiological processes underlying mTBI recovery. By integrating these diverse data sources, researchers can construct robust prognostic models that consider the multi-faceted nature of mTBI and its effects on cognitive function.

As the pivotal role IGF-1 in mTBI recovery becomes increasingly evident, future studies should explore the potential of IGF-1-related exosomes as integrated biomarkers and therapeutic agents in the context of mTBI rehabilitation. Exosomes, acting as carriers of bioactive molecules, hold tremendous promise for shedding light on the intricate genetic and neurometabolic alterations occurring post-mTBI.^27^ Their remarkable capacity to traverse the blood–brain barrier without provoking immunogenic responses opens new horizons for precise drug delivery and non-invasive monitoring of mTBI pathophysiology.^27,28^ Simultaneously, the multi-faceted actions of IGF-1 in neurogenesis, synaptic function, and neuroprotection underscore its potential as a key regulator in cognitive recovery post-mTBI.^8,10,18–20^ Moreover, the intriguing findings regarding IGF-1R-containing exosomes, as demonstrated in the context of acute kidney injury, highlight a potential avenue for treating mTBI patients.

These exosomes, enriched with IGF-1R mRNA, may enhance the sensitivity of brain cells to locally produced IGF-1, thereby promoting neuroprotection, cellular proliferation, and, ultimately, cognitive recovery.^29^ Therefore, the evaluation of IGF-1-related exosomes holds promise for not only advancing our comprehension of mTBI, but also revolutionizing clinical care for persons affected by this prevalent form of TBI.

## Conclusion

Herein, 7 of 10 WCST raw scores significantly differed between brain injury and non-brain-injury groups after adjusting by age and education level. Six of 10 WCST raw scores were associated with IGF-1 level after adjusting by age and education level. In conclusion, the current study offers valuable insights into the potential role of IGF-1 and demographic factors as prognostic markers in mTBI. However, future research should incorporate longitudinal designs, multi-modal assessments, and machine learning techniques to advance the field and translate these findings into clinical practice. By investing in rigorous and innovative research, we can bridge the current knowledge gaps and optimize the care and management of persons affected by mTBI.

## Transparency, Rigor, and Reproducibility Summary

The study was pre-registered at clinicaltrial.gov (NCT01266356). The analysis plan was not formally pre-registered. This is an observational study, and the statistical power and sample size did not calculate. Two hundred ninety-five patients and 200 healthy control participants were obtained. Participants were not told the results of their prognostic assessment. Final clinical outcome assessments and adjudications were performed by team members blinded to relevant characteristics of the participants. All surveys and questionnaires used to develop prognostic models are available from the authors. The key inclusion criteria and outcome evaluations are established standards. No replication or external validation studies have been performed or are planned/ongoing at this time, to our knowledge. Deidentified data from this study are not available in a public archive. Deidentified data from this study will be made available (as allowable according to institutional IRB standards) by e-mailing the corresponding author. Analytical code used to conduct the analyses presented in this study are not available in a public repository. They may be available by e-mailing the corresponding author The authors agree or have agreed to publish the manuscript using the Mary Ann Liebert Inc. “Open Access” option under the appropriate license.

### Availability of Data and Materials

The data that support the findings of this study are available from the corresponding author upon reasonable request.

## Abbreviations Used

AD: Alzheimer's disease
Akt: protein kinase B
CI: confidence interval
CREB: cAMP-responsive element-binding protein
IGF-1: insulin-like growth factor 1
IGF-1R: IGF-1 receptor
mTBI: mild traumatic brain injury
PI3K: phosphoinositide 3-kinase
SD: standard deviation
TBI: traumatic brain injury
TMT: Trail Making Test
WCST: Wisconsin Card Sorting Test
